# Comparison analysis of PD-1/PD-L1 inhibitors plus lenvatinib or gemcitabine/cisplatin as first-line treatment for patients with advanced intrahepatic cholangiocarcinoma

**DOI:** 10.3389/fonc.2023.1204486

**Published:** 2023-08-18

**Authors:** Jia-Xin Huang, Bo Liu, Yu Li, Xi Li, Li-Juan Ding, Nan-Ya Wang

**Affiliations:** Cancer Center, The First Hospital of Jilin University, Changchun, Jilin, China

**Keywords:** intrahepatic cholangiocarcinoma, PD-1/L1, lenvatinib, gemcitabine plus cisplatin, advanced cancer

## Abstract

**Background:**

Intrahepatic cholangiocarcinoma (ICC) is a highly aggressive primary liver cancer, with increasing incidence worldwide. Effective first-line treatments for advanced ICC patients are currently limited. Therefore, our study aimed to assess the efficacy and safety of programmed death-1 (PD-1)/programmed death-ligand 1 (PD-L1) inhibitors in combination with gemcitabine/cisplatin (GC) and lenvatinib as first-line treatment in advanced ICC patients.

**Methods:**

This retrospective cohort study included 51 advanced ICC patients, among whom 25 patients were administered with PD-1/PD-L1 plus lenvatinib and 26 patients were administered with PD-1/PD-L1 plus GC. Baseline characteristics including demographic information, medical history, clinical characteristics, laboratory data, and imaging examination were collected. The primary endpoints were progression-free survival (PFS) and sixth- and ninth-month overall survival (OS) rate. Survival curve was plotted by the Kaplan–Meier method. A Cox proportion risk model was performed to investigate independent risk factors of PFS and OS. The secondary outcomes were objective response rate (ORR), disease control rate (DCR), and adverse events.

**Results:**

The median age of advanced ICC patients in our study was 58.0 (95% confidence interval [95% CI] = 48.0–72.4) years, with 33 male and 18 female patients. Patients in the PD-1/PD-L1 inhibitors plus lenvatinib group were more likely to be in ECOG grade above 1, develop ascites, and have an elevated level of ALT. The ORR was 16.0% in the PD-1/PD-L1 inhibitors plus lenvatinib group and 23.1% in the GC group (*p* = 0.777). The DCR was 52.0% in the lenvatinib group and 46.2% in the GC group (*p* = 0.676). The combination treatment of PD-1/PD-L1 inhibitors plus lenvatinib was associated with longer PFS than the GC group; however, it was not statistically significant (lenvatinib: 9.5 months, GC: 5.1 months, *p* = 0.454). The sixth-month and ninth-month OS rates were 82.0% and 76.9% in the lenvatinib group and 87.4% and 71.5% in the GC group. After adjusting for confounders, multivariate Cox regression analysis showed that ECOG grade above 1 was an independent risk factor for PFS (hazard ratio [HR] = 3.388, 95% CI = 1.312–8.746, *p* = 0.012) and OS (HR = 4.220, 95% CI = 1.131–15.742, *p* = 0.032).

**Conclusion:**

PD-1/PD-L1 inhibitors in combination with lenvatinib or GC all demonstrated significant efficacy and safety as first-line treatment in patients with advanced ICC. As for patients who refuse or are intolerant to chemotherapy, PD-1/PD-L1 plus lenvatinib would be recommended.

## 1 Introduction

Intrahepatic cholangiocarcinoma (ICC), derived from intrahepatic bile duct, ranks as the second most common malignant tumor in liver (1, 2). The incidence of ICC has been steadily increasing worldwide, from 0.1 cases per 100,000 to 0.6 per 100,000 over the past 30 years (3, 4). The most common risk factors for ICC in Jilin Province, China are chronic hepatitis B and C infections, as well as exposure to environment toxins such as aflatoxin (5). ICC patients are often diagnosed at an advanced stage due to non-specific symptom and high malignant aggressiveness, which makes anti-cancer treatments challenging (6). Notably, only 22% of ICC patients are eligible for curative surgery, owing to delayed diagnosis. The high recurrence and metastasis rates also negatively affect post-surgery survival rates (7, 8). The development of effective systemic therapies is needed for advanced ICC patients. Gemcitabine plus cisplatin (GC) is currently the standard first-line chemotherapy for advanced ICC patients worldwide. However, its efficacy is not satisfactory as expected, given that the progression-free survival (PFS) and overall survival (OS) of GC treatment are only 8.0 and 11.7 months, respectively (9). A meta-analysis involving seven published clinical trials also showed that gemcitabine-based regimens were associated with increased adverse events (8). The poor performance status of advanced ICC patients due to frequent biliary obstruction and infection may cause chemotherapy intolerance. Therefore, identifying more effective and safer anti-cancer treatments for advanced ICC patients is imperative.

In recent years, immunotherapy such as programmed cell death 1 (PD-1) and programmed cell death ligand (PD-L1) inhibitors has shown clinical success in treating a range of solid tumors, particularly advanced cancer patients (10). Many previous studies have investigated the efficacy of immunotherapy alone or in combination with other agents in ICC patients. A multicenter phase III randomized controlled clinical study reported that compared with chemotherapy in combination with placebo, biliary tract cancer patients receiving durvalumab combined with GC showed significantly improved median OS (11.5 *vs*. 12.8 months, p = 0.021) and PFS (5.7 *vs*. 7.2 months, p = 0.001) (11). These findings suggest a promising landscape of chemotherapy combined with immunotherapy as first-line treatment in biliary tract system tumors. Nonetheless, as mentioned previously, the poor performance status of advanced ICC patients may influence their response to chemotherapy. A standard first-line anti-cancer treatment for advanced ICC patients who refuse or are intolerant to chemotherapy is urgently required.

Lenvatinib is a multi-targeted inhibitor that suppresses vascular endothelial growth factor receptor (VEGFR) 1–3, fibroblast growth factor receptor (FGFR) 1–4, platelet-derived growth factor receptor (PDGFR)α, and proto-oncogenes RET and KIT (12). Preclinical data show that lenvatinib decreases the number of tumor-associated macrophages, thereby affecting antitumor immune responses and leading to increased efficacy of PD-L1 inhibition (13). In a recent study, Dr. Zhao tested the efficacy and safety of pembrolizumab plus lenvatinib as a second-line treatment for advanced biliary tract cancer (14). The trial showed an objective response rate (ORR) of 25% and a disease control rate (DCR) of 78.1%. Notably, no grade 5 adverse events were reported and only 59.3% of patients suffered from grade 3 treatment-related adverse events. This trial manifests the efficacy and safety of immune checkpoint inhibitors (ICIs) combined with lenvatinib. However, data on immunotherapy in combination with lenvatinib as the first-line treatments for advanced ICC patients in the real world are limited.

The present study aimed to compare the efficacy and safety of PD-1/PD-L1 in combination with GC regimen and lenvatinib as the first-line anti-cancer treatment in advanced ICC cancer patients, which may provide guidance in clinical practice.

## 2 Materials and methods

### 2.1 Study population and design

This retrospective cohort study included ICC patients from February 2020 to September 2022 at the Oncology Center of the First Hospital of Jilin University. Data from the first admission were analyzed for patients with multiple admissions. Inclusion criteria were as follows: (a) age≥18; (b) histopathological or cytological diagnosis of advanced or unresectable ICC; (c) Eastern Cooperative Oncology Group (ECOG) grade of 0–2; (d) experiencing PD-1/PD-L1 inhibitors combined with lenvatinib or GC treatments over 3 weeks; (e) at least one assessable lesion according to response evaluation criteria in solid tumors (RECIST 1.1); and (f) receiving at least one efficacy assessment by enhanced computer tomography (CT) or magnetic resonance imaging (MRI). Exclusion criteria were as follows: (a) a concurrence of malignant carcinoma in other systems; (b) post-surgery of organ translation; (c) serve comorbidities such as hepatitis, heart diseases, uncontrolled epilepsy, central nervous diseases, and mental disorders; (d) incomplete clinical data; and (e) loss of follow-up. A total of 51 patients were finally analyzed in our study. This study was carried out in accordance with the principles outlined in the Declaration of Helsinki and was approved by the ethics committees of the First Hospital of Jilin University.

### 2.2 Treatment and dosing

PD-1/PD-L1 inhibitors included pembrolizumab (200 mg once every 3 weeks), sintilimab (200 mg once every 3 weeks), toripalimab (240 mg once every 3 weeks), camrelizumab (200 mg once every 2 weeks), atezolizumab (1,200 mg once every 3 weeks), and durvalumab (1,500 mg once every 3 weeks) that were administered intravenously on day 1 of the treatment cycle. The chemotherapy regimen was gemcitabine combined with cisplatin (GC regimen). Patients using the GC regimen received gemcitabine (1.0 g/m^2^) and cisplatin (25 mg/m^2^) intravenously on days 1 and 8, every 21 days for one cycle of chemotherapy. The lenvatinib group calculated the dose of oral lenvatinib use based on patient body weight: body weight < 60 kg, 8 mg/day oral; ≥ 60 kg, 12 mg/day. Every 21 days is one cycle until disease progression, death, or intolerable toxicity. Dosage delay and adjustment were all allowed.

### 2.3 Collection of baseline characteristics

We have well-trained personnel to collect the baseline characteristics of all patients. Demographic information included age, sex, weight, and height. Medical history including hepatitis and liver cirrhosis was also collected. Pathological type, the Eastern Cooperative Oncology Group performance status (ECOG-PS), physical examination, and vital signs were applied to assess clinical characteristics. Laboratory data included blood routine, blood biochemistry, liver function tests, coagulation routine, tumor markers, and urine routine. The longest diameter of targeted lesion was measured by imaging examination. All pathologic staging was defined according to the eighth edition of the American Joint Committee on Cancer TNM staging system.

### 2.4 Follow-up and outcomes

We followed patients regularly by telephone investigation, outpatient visit, and periodical reexamination. The primary outcomes were PFS, sixth-month OS rate, and ninth-month OS rate. PFS and OS were defined as the interval from the first assessment to the date of progression and death, respectively. The secondary outcomes were objective response rate (ORR), disease control rate (DCR), and adverse events. The ORR was calculated as the sum of complete response (CR) and partial response (PR) divided by total cases. The DCR was calculated as the sum of stable disease, CR, and PR divided by total cases. Objective efficacy evaluation was performed according to response evaluation criteria in solid tumors (RECIST 1.1). The adverse reactions were evaluated according to the common terminology criteria for adverse events (CTCAE) version 5.0.

### 2.5 Statistical analysis

All statistical analyses were performed using SPSS 26.0. Continuous variables were expressed as median (interquartile range) and compared by Mann–Whitney *U* test. Categorical variables were expressed as absolute number or percentage and compared by *χ* ^2^ test or Fisher’s exact test. The Kaplan–Meier method was performed to plot survival curves, with difference compared by the log-rank test. Univariate and multivariate Cox regression analyses were used to investigate independent risk factors of OS and PFS, with hazard ratios (HRs) and 95% confidence intervals (CIs). A two-tailed *p*-value < 0.05 was considered to be statistically significant.

## 3 Results

### 3.1 Baseline characteristics of patients

**Figure 1:**
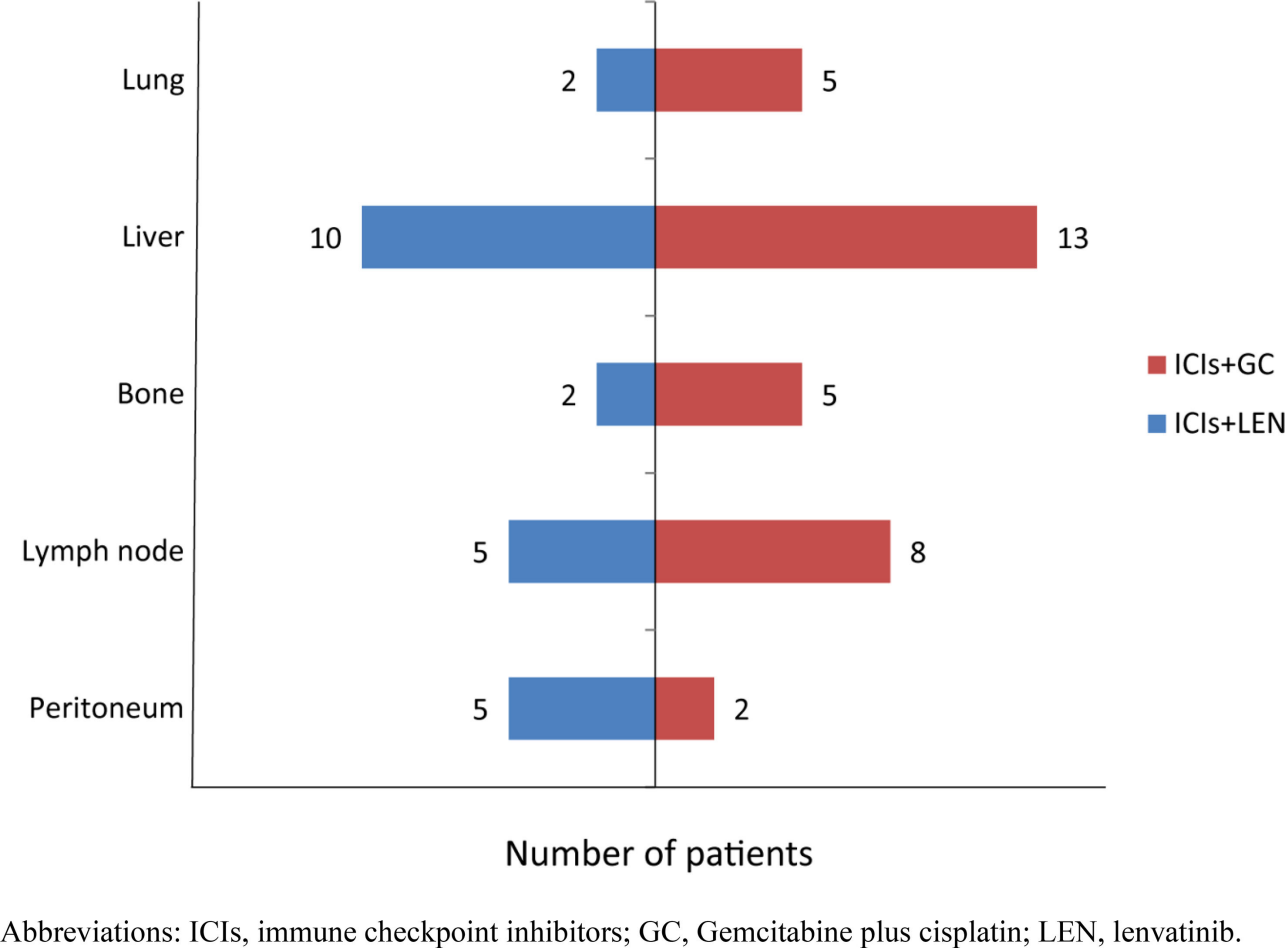
The number of patients with metastasis in various sites. ICIs, immune checkpoint inhibitors; GC, gemcitabine plus cisplatin; LEN, lenvatinib.

**Figure 2:**
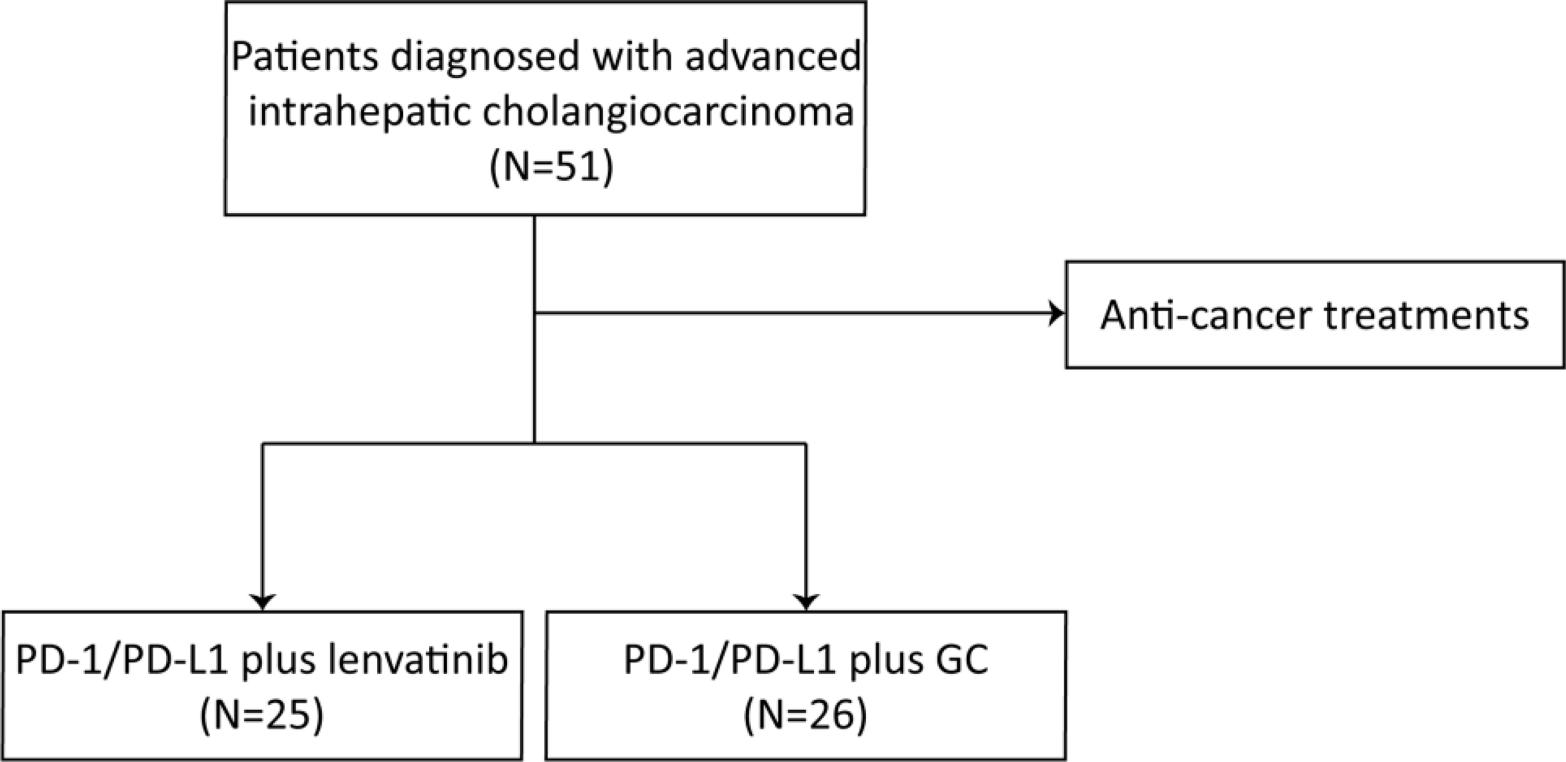
Flowchart of the present study.

A total of 51 advanced ICC cancer patients were enrolled in our study, with 33 male and 18 female patients. The number of patients aged 60 and above was 23 (45.1%). There were 44 patients classified as Child–Pugh A and 7 patients as Child–Pugh B. Seven patients had a history of liver cirrhosis and 40 patients experienced metastasis (**Figure 1**). Twenty-five (49.0%) ICC patients had poor differentiation, 10 (19.6%) had moderate differentiation, and only 1 patient had well differentiation. Patients were classified into the PD-1/PD-L1 inhibitors combined with lenvatinib group (25 cases) and the PD-1/PD-L1 inhibitors combined with GC group (26 cases) (**Figure 2**). Demographic, clinical, and follow-up information was virtually balanced between two groups except ECOG grade, ascites, and alanine transaminase (ALT) (**Table 1**). Seven (28.0%) patients in the lenvatinib group had an ECOG grade above 1, while all patients in the GC group had an ECOG grade of 0 to 1 (*p* = 0.012). The number of patients with ascites was 7 (28.0%) in the lenvatinib group, significantly higher than that of the GC group (*p* = 0.047). The value of ALT was 21.7 U/L (95% CI = 13.4–27.1) in the lenvatinib group and 26.8 U/L (95% CI = 20.2-37.5) in the GC group, with the *p*-value of 0.017.

**Table 1:** Baseline characteristics of enrolled patients.

Variables	Total (*n* = 51)	ICIs+Lenvatinib (*n* = 25)	ICIs+GC (*n* = 26)	*p*-value
Age, years				0.331
<60	28 (54.9%)	12 (48.0%)	16 (61.5%)	
≥ 60	23 (45.1%)	13 (52.0%)	10 (38.5%)	
Gender				0.918
Male	33 (64.7%)	16 (64.0%)	17 (65.4%)	
Female	18 (35.3%)	9 (36.0%)	9 (34.6%)	
ECOG				0.012*
0–1	44 (86.3%)	18 (72.0%)	26 (100.0%)	
2	7 (13.7%)	7 (28.0%)	0	
Ascites				0.047*
Yes	8 (15.7%)	7 (28.0%)	1 (3.8%)	
No	43 (84.3%)	18 (72.0%)	25 (96.2%)	
Hepatic cirrhosis				0.125
Yes	9 (17.6%)	7 (28.0%)	2 (7.7%)	
No	42 (82.4%)	18 (72.0%)	24 (92.3%)	
Metastasis				0.789
Yes	40 (78.4%)	20 (80.0%)	20 (76.9%)	
No	11 (21.6%)	5 (20.0%)	6 (23.1%)	
CA199, U/ml				0.687
<37.00	13 (25.5%)	7 (28.0%)	6 (23.1%)	
≥37.00	38 (74.5%)	18 (72.0%)	20 (76.9%)	
Child–Pugh class				0.202
A	44 (86.3%)	20 (80.0%)	24 (92.3%)	
B	7 (13.7%)	5 (20.0%)	2 (7.7%)	
ALBI score				0.811
1	13 (25.5%)	6 (24.0%)	7 (26.9%)	
2	38 (74.5%)	19 (76.0%)	19 (73.1%)	
TNM stage				0.503
III	9 (17.6%)	3 (12.0%)	6 (23.1%)	
IV	42 (82.4%)	22 (88.0%)	20 (76.9%)	
Tumor differentiation				0.505
Well	1 (2.0%)	1 (4.0%)	0	
Moderately	10 (19.6%)	4 (16.0%)	6 (23.1%)	
Poorly	25 (49.0%)	11 (44.0%)	14 (53.8%)	
Unknown	15 (29.4%)	9 (36.0%)	6 (23.1%)	
Longest diameter of tumor	5.2 (3.13–8.03)	5.1 (3.3–8.6)	6.0 (2.9–8.0)	0.950
Hemoglobin, g/L	124.0 (107.0–143.0)	130.0 (106.0–143.5)	116.5 (106.5–143.8)	0.540
Platelet, ×g/L	188.0 (140.0–231.0)	187.0 (117.0–256.0)	192.0 (144.5–222.8)	0.618
Prothrombin time, s	11.9 (11.3–12.8)	12.1 (11.3–12.9)	11.8 (11.4–12.7)	0.836
INR	1.01 (0.97–1.08)	1.02 (0.96–1.10)	1.01 (0.97–1.07)	0.618
Tbil, μmol/L	13.1 (9.5–17.5)	13.5 (8.9–18.9)	12.7 (9.6–16.8)	0.720
Albumin, g/L	37.1 (33.5–39.2)	36.2 (32.5–39.3)	37.5 (34.0–39.2)	0.540
ALT, U/L	25.1 (16.6–29.6)	21.7 (13.4–27.1)	26.8 (20.2–37.5)	0.017*
AST, U/L	29.6 (24.3–37.0)	27.7 (22.6–35.7)	30.5 (27.3–44.6)	0.134

ICIs, immune checkpoint inhibitors; GC, gemcitabine plus cisplatin; ECOG, Eastern Cooperative Oncology Group; CA199, carbohydrate antigen 199; ALBI, albumin–bilirubin; TNM, tumor/node/metastasis; INR, international normalized ratio; Tbil, total bilirubin; ALT, alanine transaminase; AST, aspartate transaminase.

*P value of < 0.05 was considered statistically significant.

### 3.2 Objective efficacy evaluation of tumor between two groups

In the group receiving PD-1/PD-L1 inhibitors in combination with lenvatinib, there was no patient experiencing CR, 4 (16.0%) patients experiencing PR, 9 (36.0%) patients experiencing SD, and 12 (48.0%) patients experiencing PD. The figure for patients receiving PD-1/PD-L1 inhibitors combined with GC was 1 (3.8%), 5 (19.2%), 6 (23.1%), and 14 (53.8%), respectively. The ORR was 16.0% in the lenvatinib group and 23.1% in the GC group (*p* = 0.777). The DCR was 52.0% in the lenvatinib group and 46.2% in the GC group (*p* = 0.676) (**Table 2**).

**Table 2:** Objective efficacy evaluation of tumor.

	Total (*n* = 51)	ICIs+Lenvatinib (*n* = 25)	ICIs+GC (*n* = 26)	*p*-value
CR, *n* (%)	1 (2.0)	0	1 (3.8)	
PR, *n* (%)	9 (17.6)	4 (16.0)	5 (19.2)	
SD, *n* (%)	15 (29.4)	9 (36.0)	6 (23.1)	
PD, *n* (%)	26 (51.0)	12 (48.0)	14 (53.8)	
ORR, *n* (%)	10 (19.6)	4 (16.0)	6 (23.1)	0.777
DCR, *n* (%)	25 (49.0)	13 (52.0)	12 (46.2)	0.676

ICIs, immune checkpoint inhibitors; GC, gemcitabine plus cisplatin; CR, complete response; PR, partial response; SD, stable disease; PD, progression disease; ORR, objective response rate; DCR, disease control rate.

### 3.3 Survival analysis

**Figure 3:**
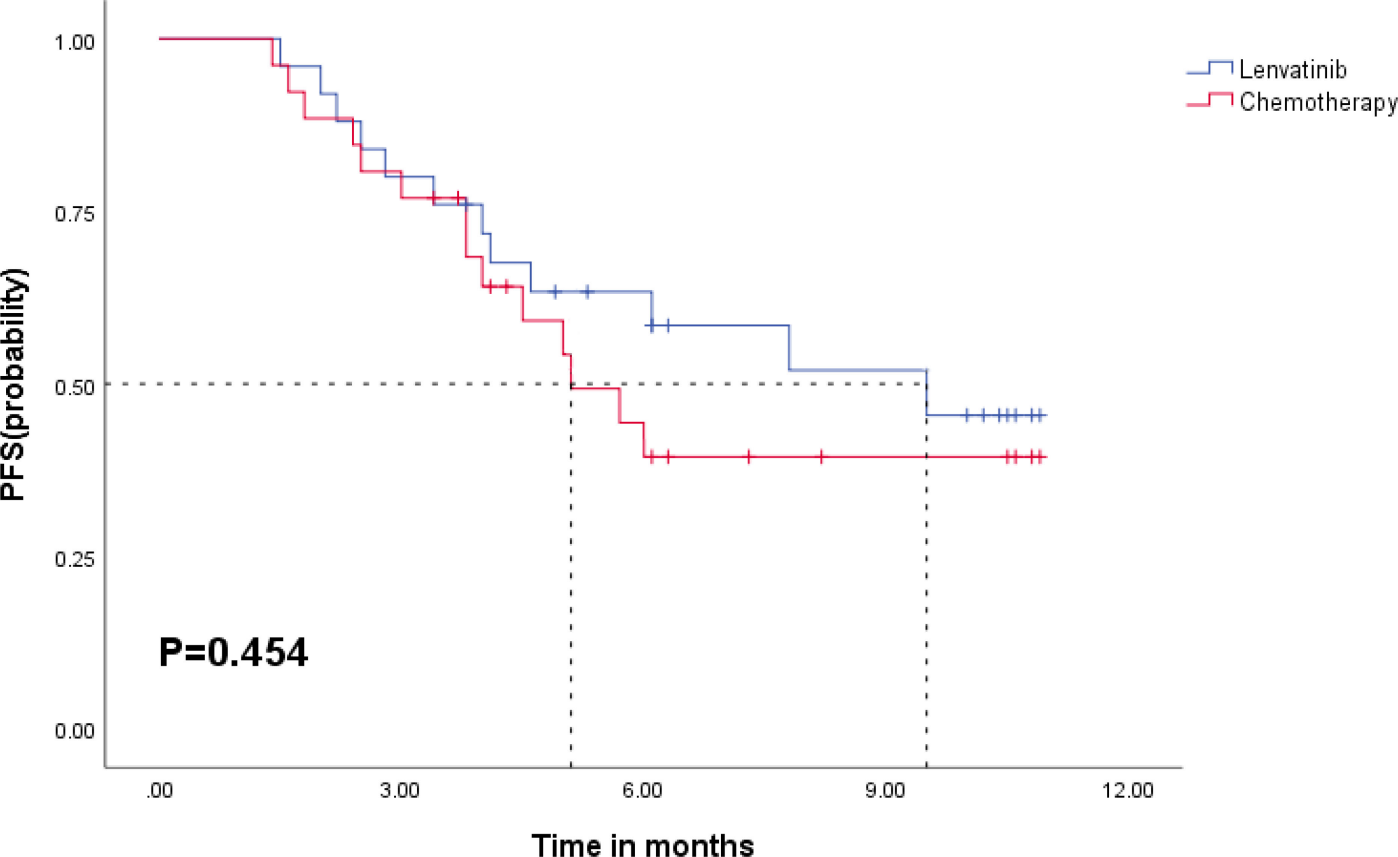
Kaplan–Meier curves for PFS in the lenvatinib group and chemotherapy group.

**Figure 4:**
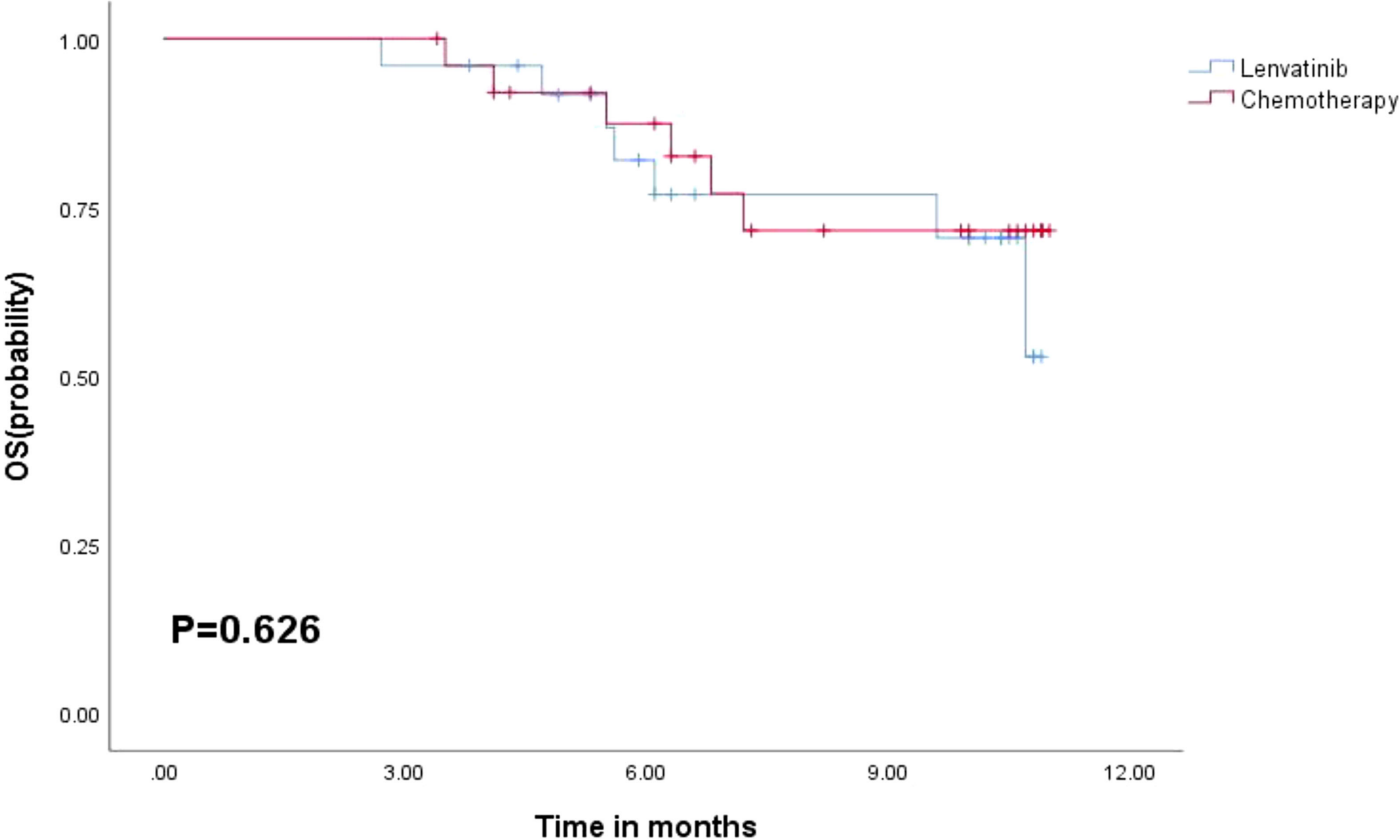
Kaplan–Meier curves for OS in the lenvatinib group and chemotherapy group.

The median follow-up was 10.2 months (95% CI = 9.7–10.7), during which 13 death cases were recorded. The median PFS of all patients was 6.1 months (95% CI = 1.5–10.7), while the median OS was not calculated due to short follow-up. The combination treatment of PD-1/PD-L1 inhibitors and lenvatinib was associated with longer PFS than the GC group, although it was not statistically significant (lenvatinib: 9.5 months, GC: 5.1 months, *p* = 0.454) (**Figure 3**). Similarly, no significant difference was observed in OS between the two groups (**Figure 4**). The sixth-month and ninth-month OS rates were 82.0% and 76.9% in the lenvatinib group and 87.4% and 71.5% in the GC group. In univariate Cox analysis, an ECOG grade above 1 or an increased level of AST was associated with shorter PFS. An ECOG grade above 1, Child–Pugh B, or an increased level of total bilirubin (Tbil) was associated with shorter OS. After adjusting for confounders, multivariate Cox regression analysis showed that ECOG grade above 1 was an independent risk factor for PFS (HR = 3.388, 95% CI = 1.312–8.746, *p* = 0.012) and OS (HR = 4.220, 95% CI = 1.131–15.742, *p* = 0.032) (**Tables 3** and **4**).

**Table 3:** Univariate and multivariate Cox analysis associated with PFS.

Variables	Univariate		Multivariate		
	Hazard ratio (95% CI)	*p*-value	Hazard ratio (95% CI)		*p*-value
Age, years					
≤60	Reference				
>60	1.06 (0.49–2.30)	0.885			
Gender					
Female	Reference				
Male	0.97 (0.44–2.14)	0.930			
ECOG					
0–1	Reference				
2	3.00 (1.19–7.57)	0.020*	3.39 (1.31–8.75)	0.012*	
Ascites					
No	Reference				
Yes	0.58 (0.17–1.95)	0.380			
Hepatic cirrhosis					
No	Reference				
Yes	1.12 (0.42–2.97)	0.823			
Metastasis					
No	Reference				
Yes	1.32 (0.53–3.31)	0.552			
CA199, U/ml					
<37.00	Reference				
≥37.00	1.34 (0.54–3.35)	0.528			
Child–Pugh class					
A	Reference				
B	1.18 (0.41–3.44)	0.761			
ALBI score					
1	Reference				
2	1.50 (0.60–3.75)	0.386			
TNM stage					
III	Reference				
IV	1.37 (0.47–3.99)	0.567			
Treatment					
ICIs+GC	Reference				
ICIs+ lenvatinib	0.75 (0.34–1.62)	0.457			
Longest diameter of tumor	0.98 (0.85–1.13)	0.782			
Hemoglobin, g/L	1.01 (0.99–1.03)	0.408			
Platelet, ×g/L	1.00 (1.00–1.00)	0.722			
Prothrombin time, s	0.85 (0.61–1.17)	0.319			
INR	0.15 (0.00–6.70)	0.323			
Tbil, μmol/L	1.02 (1.00–1.04)	0.117			
Albumin, g/L	1.00 (0.92–1.09)	0.980			
ALT, U/L	1.00 (0.99–1.01)	0.673			
AST, U/L	1.01 (1.00–1.03)	0.095	1.02 (1.00–1.03)	0.051	

Those variables found significant at P < 0.1 in the univariate analyses were entered into the multivariate Cox regression analyses.

CI, confidence interval; ICIs, immune checkpoint inhibitors; GC, gemcitabine plus cisplatin; ECOG, Eastern Cooperative Oncology Group; CA199, carbohydrate antigen 199; ALBI, albumin–bilirubin; TNM, tumor/node/metastasis; INR, international normalized ratio; Tbil, total bilirubin; ALT, alanine transaminase; AST, aspartate transaminase.

*P value of < 0.05 was considered statistically significant.

**Table 4:** Univariate and multivariate Cox analysis associated with OS.

Variables	Univariate		Multivariate	
	Hazard ratio (95%CI)	*p*-value	Hazard ratio (95%CI)	*p*-value
Age, years				
≤60	Reference			
>60	1.90 (0.62–5.84)	0.260		
Gender				
Female	Reference			
Male	1.31 (0.40–2.25)	0.659		
ECOG				
0–1	Reference			
2	5.26 (1.71–16.22)	0.004*	4.22 (1.13–15.74)	0.032*
Ascites				
No	Reference			
Yes	0.87 (0.19–3.96)	0.859		
Hepatic cirrhosis				
No	Reference			
Yes	1.83 (0.50–6.70)	0.359		
Metastasis				
No	Reference			
Yes	2.28 (0.50–10.35)	0.287		
CA199, U/ml				
<37.00	Reference			
≥37.00	1.95 (0.43–8.81)	0.384		
Child–Pugh class				
A	Reference			
B	3.55 (1.08–11.60)	0.036*	2.27 (0.41–12.60)	0.347
ALBI score				
1	Reference			
2	2.66 (0.59–12.06)	0.203		
TNM stage				
III	Reference			
IV	3.00 (0.39–23.12)	0.291		
Treatment				
ICIs+GC	Reference			
ICIs+ lenvatinib	1.31 (0.44–3.91)	0.628		
Longest diameter of tumor	1.01 (0.82–1.25)	0.909		
Hemoglobin, g/L	0.99 (0.96–1.02)	0.471		
Platelet, ×g/L	1.00 (0.99–1.00)	0.252		
Prothrombin time, s	0.80 (0.49–1.31)	0.382		
INR	0.20 (0.00–44.30)	0.562		
Tbil, μmol/L	1.03 (1.00–1.05)	0.025*	1.00 (0.97–1.03)	0.915
Alb, g/L	0.93 (0.82–1.05)	0.231		
ALT, U/L	0.99 (0.97–1.02)	0.549		
AST, U/L	1.00 (0.98–1.03)	0.718		

Those variables found significant at p < 0.1 in the univariate analyses were entered into the multivariate Cox regression analyses.

CI, confidence interval; ICIs, immune checkpoint inhibitors; GC, gemcitabine plus cisplatin; ECOG, Eastern Cooperative Oncology Group; CA199, carbohydrate antigen 199; ALBI, albumin–bilirubin; TNM, tumor/node/metastasis; INR, international normalized ratio; Tbil, total bilirubin; ALT, alanine transaminase; AST, aspartate transaminase.

*P value of < 0.05 was considered statistically significant.

### 3.4 Comparison of safety between two combination treatments

No grade 4 adverse event and related death was recorded in both groups. There were four (16.0%) grade 3 adverse events in the lenvatinib group, namely, fatigue (*n* = 1), hand–foot skin response (*n* = 1), appetite loss (*n* = 1), and dizziness (*n* = 1). There were four (15.4%) grade 3 adverse events in GC group, namely, fatigue (*n* = 1) and myelosuppression (*n* = 3) (**Table 5**). In the lenvatinib group, the adverse events were mainly gastrointestinal side effects such as decreased appetite, nausea and vomiting, constipation, and diarrhea. In the GC group, the most common chemotherapy-related adverse events were fatigue, decreased appetite, nausea and vomiting, and myelosuppression. All clinical presentations were alleviated by reducing drug dose.

**Table 5:** Treatment-related adverse events in the present study.

Adverse events n (%)	All patients (*n* = 51)		ICIs+Lenvatinib (*n* = 25)		ICIs+GC (*n* = 26)	
	Grades 1–2	Grade 3	Grades 1–2	Grade 3	Grades 1–2	Grade 3
Fatigue	16 (31.4)	2 (3.9)	7 (28.0)	1 (4.0)	9 (34.6)	1 (3.8)
Hypertension	3 (5.9)	0	3 (12.0)	0	0	0
Skin itching	9 (17.6)	0	7 (28.0)	0	2 (7.7)	0
Skin eruption	6 (11.8)	0	5 (20.0)	0	1 (3.8)	0
Hand–foot skin reaction	2 (3.9)	1 (2.0)	2 (8.0)	1 (4.0)	0	0
Loss of appetite	20 (39.2)	1 (2.0)	8 (32.0)	1 (4.0)	12 (46.2)	0
Nausea and vomiting	16 (31.4)	0	7 (28.0)	0	9 (34.6)	0
Constipation	8 (15.7)	0	4 (16.0)	0	4 (15.4)	0
Diarrhea	9 (17.6)	0	5 (20.0)	0	4 (15.4)	0
Dizzy	6 (11.8)	1 (2.0)	3 (12.0)	1 (4.0)	3 (11.5)	0
Myelosuppression	12 (23.5)	3 (5.9)	1 (4.0)	0	11 (42.3)	3 (11.5)
Transaminase elevation	5 (9.8)	0	2 (8.0)	0	3 (11.5)	0
Hypothyroidism	3 (5.9)	0	2 (8.0)	0	1 (3.8)	0
Immune hepatitis	1 (2.0)	0	1 (4.0)	0	0	0

ICIs, immune checkpoint inhibitors; GC, gemcitabine plus cisplatin.

## 4 Discussion

Owing to delayed diagnosis, ICC patients are always diagnosed at an advanced stage, inappropriate for curative surgery and with poor prognosis. Adjuvant systemic treatment will be recommended after surgery resection or in the palliative care. GC is currently accepted as the standard first-line chemotherapy for advanced ICC patients; however, its limited efficacy and potential toxicity fail to significantly improve prognosis. The occurrence of immunotherapy has revolutionized treatment landscape of various solid tumors, including ICC. However, many previous studies showed that the efficacy of immunotherapy as a monotherapy was modest in advanced ICC patients (15, 16). The present study aimed to explore the efficacy and safety of combination treatments based on PD-1/PD-L1 in advanced ICC patients. We retrospectively analyzed patients receiving PD-1/PD-L1 inhibitors combined with GC or lenvatinib. Objective tumor response and survival analysis were used to compare the efficacy between two combination treatments. Adverse events were also recorded to evaluate the safety. The results of our study showed that both ICIs combined with GC and lenvatinib may be promising first-line treatments for advanced ICC patients, with no significant prognostic difference between two groups.

Chemotherapy has been confirmed to upregulate the expression of immune checkpoint, which can result in significant modifications in immune cell infiltrate (17). Combining chemotherapy with ICIs may achieve longer survival for cancer patients. Many previous clinical studies have confirmed a better tumor response and survival of receiving PD-1/PD-1 inhibitors plus chemotherapy than standard chemotherapy in patients with non-small cell lung cancer (18), breast cancer (19), esophagus cancer (20), and biliary tract cancer (11). The results of our study revealed that PD-1/PD-L1 inhibitors plus GC treatment had an ORR and DCR of 23.1% and 46.2%, respectively, in advanced ICC patients, with 6-month and 9-month OS rates of 87.4% and 71.5%, respectively. Similarly, in a phase II trial, camrelizumab plus gemcitabine plus oxaliplatin (GEMOX) has shown a promising antitumor activity and acceptable safety profile as first-line treatment in advanced biliary tract cancer patients. Twenty (54%) out of 37 patients had an objective response. The median PFS was 6.1 months and the median OS was 11.8 months, respectively (21). Moreover, some studies also indicated that PD-1 inhibitors can resensitize biliary tract cancer to chemotherapy. In a phase II study, patients treated primarily with gemcitabine- or cisplatin-based chemotherapy were subsequently treated with nivolumab plus gemcitabine and cisplatin. Some patients showed a CR, and one showed a PR, suggesting that nivolumab is able to resensitize chemotherapy with gemcitabine and cisplatin (22).

Lenvatinib is a multiple receptor tyrosine kinase inhibitor targeting mainly vascular endothelial growth factor (VEGF) and fibroblast growth factor (FGF) receptors (12). Notably, preclinical research showed that the antitumor activity of lenvatinib will improve when combined with ICIs by reducing tumor-associated macrophages (TAMs) and increasing the percentage of activated CD8+ T cells secreting interferon (IFN)-γ+ and granzyme B (GzmB) (23). Wang et al. reported that the ORR was 12% (95% CI: 1.7–22.7), with a median PFS of 3.8 months (95% CI: 1.3–6.3) and an OS of 11.4 months in advanced biliary tract carcinoma patients receiving lenvatinib as first-line therapy (24). Compared with lenvatinib alone, our study showed a higher ORR (16.0%) and longer median PFS (9.5 months) of lenvatinib plus PD-1/PD-L1 inhibitors. Consistent with our results, the median PFS of lenvatinib plus PD-1/PD-L1 inhibitors from a recent study was 8.63 months in ICC patients, longer than lenvatinib alone though slightly shorter than the results of our study (25). In the same trend, Xie et al. recorded a median PFS of 5.83 months, with an ORR and DCR of 17.5% and 75.0% respectively (26). In a phase II study, the ORR and DCR reached 42.1% and 76.3%, respectively, in unresectable biliary tract cancer patients receiving lenvatinib plus PD-1 inhibitors as first-line treatment (27). Zhu et al. recently reported that PD-1/PD-L1 inhibitors plus chemotherapy combined with lenvatinib represented an effective and tolerable treatment option in patients with advanced biliary tract cancer (28). One possible reason why their results were significantly better than ours is because of the difference in patients’ baseline profile. Moreover, there may be a difference in the efficacy of targeted therapy combined with immunotherapy and chemotherapy between patients with gallbladder cancer, ICC, and extrahepatic cholangiocarcinoma (29). Although there are mild differences from various studies, it is apparent that the combination of lenvatinib with PD-1/PD-L1 could be an effective treatment for advanced ICC patients.

In the current study, we found that patients receiving PD-1/PD-L1 inhibitors plus lenvatinib had a poorer physical status, had a higher ECOG grade, and had frequent presence of ascites. This may be due to the selection bias that patients would be treated with lenvatinib only when they are intolerant to chemotherapy in the clinical practice. However, no significant difference was observed in tumor objective response and survival between patients receiving PD-1/PD-L1 inhibitors plus GC and lenvatinib. In the multivariate Cox analysis adjusting for confounding factors, only ECOG grade was the independent risk factor for PFS and OS. As for safety, the incidence of side effects was higher in the chemotherapy group, but it was not statistically significant. Moreover, no grade 4 adverse event and related death was recorded in both groups. Therefore, we speculated that PD-1/PD-L1 plus lenvatinib would be recommended as a promising first-line treatment for advanced ICC patients who refuse or are intolerant to chemotherapy. In the clinical practice, we can formulate individualized anti-cancer treatments depending on the patient’s physical performance.

This study has some limitations. First, this is a retrospective single-center study, with a small number of participants and short follow-up. The median OS was not reached in both groups. There may be type II error in our study considering that the conclusions were generated based on non-statistically significant results. A large-scale randomized controlled prospective study is needed to confirm the results of the present study. Second, we included all patients receiving PD-1/PD-L1 inhibitors plus chemotherapy or lenvatinib in this retrospective study, not differentiating specific drugs. The choice of the PD-1/PD-L1 inhibitor is highly individualized in the real world considering drug accessibility, price, medical insurance coverage, and other factors. Moreover, there is no evidence showing which PD-1/PD-L1 inhibitor is better than others in advanced ICC patients. Most patients also lacked data on PD-L1 expression testing, which may have an influence on their response to immunotherapy. Third, all patients enrolled in our study were from one medical institution in China. The efficacy and safety of PD-1/PD-L1 inhibitors plus chemotherapy and lenvatinib as the first-line treatment beyond the current region and race need to be proven in the future.

In conclusion, PD-1/PD-L1 inhibitors in combination with chemotherapy and lenvatinib are both beneficial to tumor control and survival extension as first-line treatment in advanced ICC patients, with no severe adverse events. As for patients with poor physical performance or intolerance to chemotherapy, ICIs plus lenvatinib could be recommended.

## Data availability statement

The original contributions presented in the study are included in the article/supplementary material. Further inquiries can be directed to the corresponding author.

## Ethics statement

The studies involving humans were approved by The ethics committees of the First Hospital of Jilin University. The studies were conducted in accordance with the local legislation and institutional requirements. The participants provided their written informed consent to participate in this study.

## Author contributions

All authors have read and approved the final manuscript. N-YW conceived and designed the study. J-XH, BL YL, XL, and L-JD assisted with the development of the methods. J-XH, BL, and YL performed the data analysis. J-XH and BL drafted the initial manuscript. L-JD and N-YW gave many valuable comments on the draft and polished it. All authors assisted with the interpretation of the findings, commented on drafts of the manuscript, and approved the final version.
